# Musical Enjoyment and Reward: From Hedonic Pleasure to Eudaimonic Listening

**DOI:** 10.3390/bs12050154

**Published:** 2022-05-19

**Authors:** Mark Reybrouck, Tuomas Eerola

**Affiliations:** 1 Musicology Research Group, Faculty of Arts, KU Leuven—University of Leuven, 3000 Leuven, Belgium; 2 Department of Art History, Musicology and Theatre Studies, Institute for Psychoacoustics and Electronic Music (IPEM), 9000 Ghent, Belgium; 3 Department of Music, Durham University, Durham DH1 3RL, UK; tuomas.eerola@durham.ac.uk

**Keywords:** music listening, enjoyment, reward, coping, homeostasis, hedonic, eudaimonic, adaptive/maladaptive listening

## Abstract

This article is a hypothesis and theory paper. It elaborates on the possible relation between music as a stimulus and its possible effects, with a focus on the question of why listeners are experiencing pleasure and reward. Though it is tempting to seek for a causal relationship, this has proven to be elusive given the many intermediary variables that intervene between the actual impingement on the senses and the reactions/responses by the listener. A distinction can be made, however, between three elements: (i) an objective description of the acoustic features of the music and their possible role as elicitors; (ii) a description of the possible modulating factors—both external/exogenous and internal/endogenous ones; and (iii) a continuous and real-time description of the responses by the listener, both in terms of their psychological reactions and their physiological correlates. Music listening, in this broadened view, can be considered as a multivariate phenomenon of biological, psychological, and cultural factors that, together, shape the overall, full-fledged experience. In addition to an overview of the current and extant research on musical enjoyment and reward, we draw attention to some key methodological problems that still complicate a full description of the musical experience. We further elaborate on how listening may entail both adaptive and maladaptive ways of coping with the sounds, with the former allowing a gentle transition from mere hedonic pleasure to eudaimonic enjoyment.

## 1. Introduction

The question of how music evokes pleasure and reward is a hot topic in current music research. There is a long history of narratives that try to explain why listeners are moved by music, with a special focus on the pleasurable experience of listening to sad music. But even since the emergence of music psychology as an established discipline, there are a lot of open questions that pertain to both the theoretical constructions and the gathering of empirical data in this domain.

On the theoretical level, researchers are still struggling with conceptual and terminological issues, such as the construct validity of concepts such as pleasure, reward, and enjoyment, and the distinction between hedonic pleasure and eudaimonic enjoyment. There is, quite generally, a major distinction to be made between the pleasurable experience of music listening (*hedonia*) and the happiness and the meaningful sense it provides to the self (*eudaimonia*). The hedonic experience focuses rather narrowly on the experience of pleasant feelings, with a healthy balance between a positive and negative effect. It sees pleasure as the satisfaction of homeostatic needs such as hunger, sex, and bodily comfort, as well as the avoidance of pain. Eudaimonic enjoyment, in contrast, stretches good feelings beyond immediate satisfaction. It aims toward broader goals, such as the realization of our potential and personal growth, as exemplified in an outstanding athletic event, an artistic performance, prosocial behavior, or a stimulating conversation [1,2,3]. There is, however, a multiplicity of conceptual and operational definitions of hedonia and eudaimonia, which complicate the comparisons and generalizations [4,5,6,7] (see [8] for an overview).

Several frameworks have been proposed in this regard, such as the homeostatic theory [9,10,11,12], the reward theory [13,14,15], and a position that emphasizes empathetic engagement with emotional events in terms of approach/avoidance behavior [16] (see [3] for a critical discussion). On the empirical level, a considerable amount of research has been conducted on finding the biochemical correlates of psychological reward, with a focus on specific hormonal markers. Conflicting results have been found, however, with regard to the release of hormones and neurotransmitters such as prolactin and oxytocin, as well as the mediating role of dopamine. Furthermore, attempts have been made to relate these findings with dispositional traits, such as empathy or other personality-related traits, but these results are also not yet conclusive. It seems, therefore, that this area of research must still mature to some extent, with major methodological challenges related to the assessment of the physiological and psychological mechanisms of musical pleasure and reward and their interdependencies.

**Figure 1:**
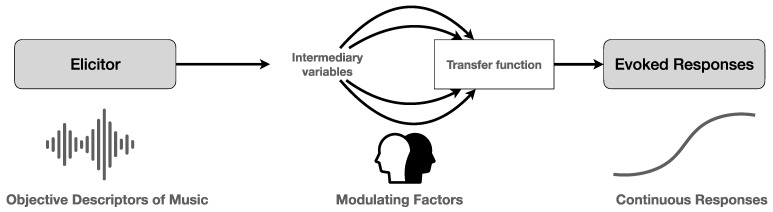
Overview of the elicitor–response schematic with modulating factors and transfer function.

A major point in this regard is the search for causal relations between music as a stimulus and its possible effects. This has proven to be somewhat elusive up to now, given the many intermediary variables that operate between the music as a possible elicitor and its evoked responses (see Figure 1). Though it is tempting to conceive of this relationship in terms of a mathematical function, with the domain as all possible acoustical features (input values), the range as all possible responses (output values), and the mapping between input and output as a transfer function, this holds only for the lower levels of sensory processing, which makes it difficult to conceive of music processing in terms of psychobiological equivalence [17,18]. Yet, it is possible to argue for an operational approach, which contains three elements: (1) an objective description of the acoustic features of the music and their possible role as elicitors; (2) a description of the possible modulating factors—both external/exogenous and internal/endogenous ones—and (3) a continuous and real-time description of the responses by the listener, both in terms of their psychological reactions and their physiological correlates.

Current research derived from psychoacoustics and music information retrieval (MIR) has provided a workable feature set of acoustical properties—the MIRToolbox [19] —which are related to the traditional dimensions of music theory (pitch and tonality, rhythm, timbre, and dynamics). Further reduction in the dimensionality of this feature set by means of Principal Component Analysis has left six major components: fullness, brightness, activity, timbral complexity, pulse clarity, and key clarity (see [20,21] for a technical description). Other models of music signal analysis have been suggested as well [22]. Such a thick description makes it possible to provide an objective description of the music—at least for Western music and emotions—and to look for possible causal relations in the generation of physiological reactions to the sounds. Especially at the lower levels of sensory processing, this has constrained the variability of psychophysical responses so as to leave open the option of conceiving of music processing in terms of quasi-linear/causal relationships. The possibility of measuring and assessing physiological reactions, on the other hand, provides another set of empirical data that can also be gathered in an objective way. An important challenge, however, is to relate these physiological correlates to conscious experience, as many of them operate at levels below deliberate consciousness and control.

**Figure 2:**
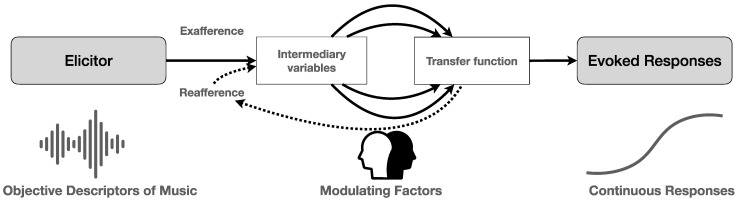
Overview of the elicitor–response schematic with dual input from self-generated (reafference) and externally generated (exafference) sources.

Another challenge, furthermore, is to map the variability of the modulating factors. Much is to be expected here from research on the dispositional traits of music listeners, such as trait empathy and openness to experience, to mention just two of them, and methods of data gathering that portray the listener’s ontogenetic development (life history, learning curve, etc.). The classical input–output mode of information processing, however, should be challenged both with regard to the linearity and causality between the processing stages. More promising is a circular model that uses the output (reactions and responses) as a new input to modify the mapping between input and output and that can alter the quality and intensity of the musical experience. Music processing, in this view, could be considered as relying on a dual input: one that stems from external stimuli (the music) and one that is self-generated and stems from somatosensory—including interoception and proprioception—and vestibular responses to the music, which is somewhat analogous to the distinction between exafferent (external stimuli) and reafferent (self-generated) stimuli (see [23,24,25,26,27] for an overview) (see Figure 2).

In this article, we provide an overview of the current and extant research on musical enjoyment and reward with special attention to some methodological problems that still complicate a full description of the musical experience. These problems are situated primarily at the output side of music processing and can be related to the assessment and measurement of responses to the musical stimuli. Not all of these are translated into overt behavior, and some of them become manifest only after some temporal delay, which makes it difficult to monitor real-time and ongoing reactions and responses to the sounds. Some measurement techniques, furthermore, are constrained with respect to their temporal and spatial resolution, which is a major issue in neuroimaging, with two major questions: “when” does a response happen and “where” is it exactly triggered in the brain? The questions are even irrelevant, to some extent, as there are no really unique triggering areas or points in the brain. The picture that is emerging in recent research points in the direction of networks, and most meta-analyses of neural correlates of, e.g., basic emotions draw such an inconsistent picture of the specific involved areas that this is not really saying much [28,29] or they mainly relate to broad brain areas [30,31]. As such, there are measurement restrictions, which constrain the scope and breadth of what can be investigated. Yet, there already exists a whole array of objectively measurable phenomena, such as bodily reactions (thrills and chills, lacrimation, piloerection), neuroendocrine reactions and their biochemical markers (dopaminergic reward system), physiological reactions (heart rate, blood pressure, respiration rate, electrodermal activity, and EMG), and neural activity (EEG, ECoG, fMRI, PET, and fNIRS).

This search for measurable phenomena has led also to a rather limited focus on typical kinds of music, such as sad music or very loud music, which have been found to play a preferential role in the generation of music-evoked bodily responses. The theoretical frameworks, however, are not yet totally coherent. Much research on bodily reactions stems from animal testing or experiments with humans outside the domain of music, which makes it difficult to translate the findings to the case of music and human listeners (see [32]). Yet, it is important to gather as many empirical data as possible in various clinical settings in an attempt to “naturalize” the musical experience, even if the final explanations remain tentative in their generalizing power (see [33] for a broad overview). The neurobiological data, which may be collected in this regard, are of decisive importance, as they provide the necessary empirical underpinnings that bring together findings from neuroaesthetics—the branch of research that studies the neural correlates of music listening or performing—psychobiology and the neurochemistry of emotions, as studied in affective neuroscience. It is hoped that, from these findings, a kind of inductive generalization should be possible, to shed light on the way listeners cope with the sounds they are listening to. Listeners, however, are not fixed and stable beings. They evolve throughout their lives, and even at certain relative stable phases in their ontogenetic development, there is still a lot of variability, due to mood changes, allocation of attention, motivational factors, learning and acculturation, and many other external conditions, all of which can have an impact on their listening experience. Furthermore, it is possible to report on this experience in a descriptive and neutral way, but it is also arguably possible to try to intervene in this experience. Listening, then, can be considered as a *learnable skill*, with the aim of enhancing the level of processing to modulate the listening experience (see, e.g., [34,35,36,37]. This can be done in an adaptive or a maladaptive way, in the sense that both the choice of the music and the style of listening may determine the ultimate effects of dealing with the sounds [33]. It means that both the music and the listener matter, and this immediately entails a major challenge as there is a huge amount of variability in the music and the dispositional machineries of individual listeners. Yet, it is feasible to argue for the achievement of a so-called aesthetic attitude in an attempt to favor the possible transition from mere “hedonic pleasure” to a full-fledged “eudaimonic experience”. In what follows, we elaborate on these terms and provide an overview of some underlying mechanisms of musical enjoyment and reward.

## 2. Music Listening: From Acoustic Processing to Full-Fledged Experience

The musical experience can be considered as a multivariable function with biological, psychological, and cultural factors as independent variables that modify the ways listeners experience the music. All these variables may contribute and interact, but none of them, taken separately, is sufficient to fully explain the final experience. The biological substrates, however, should have a preferential role in providing some of the explanatory mechanisms of musical pleasure and reward. They may be responsible for the strongest and most efficient reward experience no matter what the generator is. This does not mean, however, that they are necessarily the primary triggers of musical reward. As important are cultural factors for triggering reward.

Up to now, however, there is not yet conclusive evidence about the exact nature of the musical experience, despite the theoretical proposals and a lot of data gathering about the reward mechanisms that are involved in music listening [9]. There is, moreover, a distinction to be made between the study of the brain’s reward system at a proximal or proximate stage, which can be described mainly in terms of biochemistry, and the experience of reward at a distal or ultimate stage (see [38,39]).

Proximate factors explain why a particular organism does something. They include mechanistic explanations of how something works as well as ontogenetic or developmental explanations of how something develops in the particular lifetime of an organism (ontogeny). These explanations are abundant in the domains of (neuro)physiology and developmental biology. In addition, however, there is also the domain of ultimate factors, which is related to the longer time scale of evolution. It tries to understand how and why a particular capability or trait arose in a species with questions about phylogeny and the ultimate function or the survival value of the trait (see [40] for an explanation of the terms).

**Figure 3:**
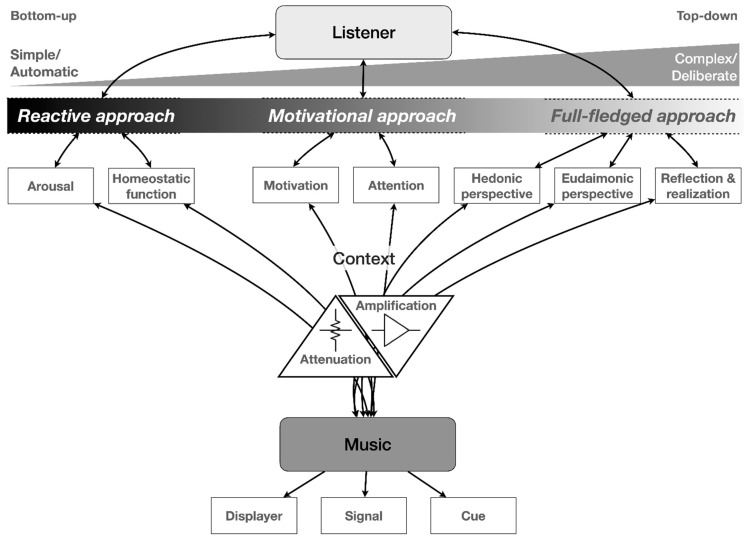
Schematic overview of the distinct levels of music processing and their interactions.

To clarify things, we take up the above-mentioned aim to naturalize the musical experience by first having a glimpse at the way listeners interact with their sonic environment, to investigate how music affects our biological systems and how it may have an emotional and cognitive effect, and we conclude with the role of the dispositional machinery of individual listeners. There is a danger, however, of some reductionism by taking a bottom-up perspective that conceives of listeners as biological machines subject to few variations related to personality traits, etc. In addition, there is also the impact of culture on emotions, and this entails a top-down approach as well. In order to provide a first global overview of this dynamic tension between levels of processing and the role of possible modulating factors, we insert a schematic overview in Figure 3, which shows a gradual transition from low-level, simple, and automatic reactions over motivational–attentional processing to a full-fledged eudaimonic experience. The figure is explained throughout the remaining text of this article.

### 2.1. Coping with the Sounds

The primary role of the hearing system is biological. Evolved as a system to recognize energy changes in the environment, it functions as a highly sensitive acoustic warning system to interpret acoustic signals and cues in terms of optimal navigation in the environment and to recognize sound sources in terms of survival and reproduction value [41,42]. It makes it possible to conceive of listening in terms of coping behavior, based on two assumptions: (i) music, as a temporal and sounding art, is a source of vibrational and transferable energy that impinges upon the senses; (ii) music can be considered as a challenging environment, in either a positive or a negative sense. Music, in that view, can be valued as a stressor or a reward, and listening can be described in terms of adaptive listening, in search of beneficial effects, or in terms of maladaptive listening, in search of mere arousal and overstimulation [43].

Coping, in its broadest definition, is a survival mechanism of living organisms in their interaction with their environment. It has been defined as the “cognitive and behavioral efforts to manage specific external and/or internal demands that are appraised as taxing or exceeding the resources of the person” [44] (p. 141). Coping behavior, however, is not merely reactive behavior to the solicitations of the environment. It can also be seen as a way of making sense of that environment, ranging from overt physical reactions, to affective-emotional reactions, to cognitive and mental operations, which, together constitute a full-fledged experience. As such, there is some analogy with the ethological distinction between the interpretation of signals and cues in the animal kingdom [45,46] (see also [47]). Signals have evolved for purposeful communicative behavior with the aim of communicating a specific message to an observer. Communication, in that case, is intentional. A cue, in contrast, is a non-purposeful artifact that is nevertheless informative but only as an unintended consequence. Signals, moreover, rely on innate behavioral and physiological mechanisms; cues, in contrast, imply learned and artefactual behaviors.

It is tempting to translate this to the realm of music, as did Huron. In what he coined the Acoustic Ethological Model (AEM), he investigated the relation between perceived acoustical features—such as pitch and intensity—and distinguished four acoustical conditions that may function as signals or cues: high pitch and high intensity, associated with fear or alarm; high pitch and low intensity, associated with appeasement or friendliness; low pitch and high intensity, associated with aggression or seriousness; and low pitch and low intensity associated with sadness, sleepiness, and relaxation [48]. It is not easy, however, to decide whether music stimuli should be considered as signals or cues. Signaling, in ethology, has as its purpose the changing of the behavior of the observing animal, as is the case, e.g., with showing submissive behavior in order to avoid the aggression of a dominant animal [49]. From an evolutionary perspective, this makes sense only if there is some benefit for the signaling animal. Signals, therefore, do not communicate primarily an animal’s or a person’s displayed emotion. Their purpose is “manipulation” rather than “information” about the emotional state of the signaler [50] (see also [51]). This is exemplified typically in sadness-related displays such as weeping, which encourage the termination of aggression as well as the evocation of feelings of prosocial compassion [52]. Signals, therefore, are more predictive of the evoked or induced effects on the observers than the emotional states of the displayers, and this also has consequences for emotion research in general, which has been mostly oriented, somewhat wrongly, to the displayers’ side [51]. Applying this to music is challenging, as listening is oriented primarily toward the receiver of the signals. To the extent, however, that music is hypothesized to express emotional or other narrative content, it can also be considered as a displayer, albeit at a virtual level [53,54]. Music, then, can be considered as a virtual person or agent.

There is, furthermore, a lot of freedom in the transition from acoustic cues to musical signals. Some acoustic features, however, have a preferential role in the context of purposive communication. Such is the case, e.g., for the prosodic elements of speech and their musical counterparts, as a kind of voice–music analog with music, emulating the acoustic features of emotional vocalizations [55,56]. This means that music and vocal prosody are hypothesized to share common affective resources, both for production and reception. Some of them have been identified in typical examples as sad speech prosody, with a reduction to six major acoustic factors: lower pitch, smaller pitch intervals, quieter sound, slower tempo, more mumbled articulation, and a darker timbre [10]. They have in common low physiological arousal. In addition, there are also other prosodic analogies, as in the emotional valence of human speech and in nonverbal vocalizations such as screams, roars, and pain cries [57]. All of these function mainly as relevant communication signals, which deviate from regular phonation by occupying a more chaotic regime in the distribution of spectral energy. They thus increase their attention-capturing and alarm-encoding power by occupying a privileged niche or restricted portion of the acoustic space that corresponds mainly to the perceptual attribute of roughness. As such, they facilitate detection by the engagement of subcortical structures in the brain, which are critical to the rapid appraisal of possible harmful stimuli and their sources [58]. This is clearly interpretable as a signal in the ethological sense, with reliance on innate behavioral and physiological coping mechanisms. Yet, it is also possible also to use these features (e.g., roughness) in some types of music to emulate the emotions that are related to fear or anger. It illustrates again the complex and multifaceted character of music and the limitations of a biased reduction of music processing to a mere ethological model. As a partial explanation, however, the model has a lot of explanatory power—especially at the low levels of processing—even if it still needs additional research.

### 2.2. Music Affects Our Biological Systems: Affective-Emotional Impact

Conceiving the hearing sense as a warning system calls forth an adaptive view on musical sense-making in the sense that listeners, as evolved biological beings, can rely on a dispositional toolkit for survival in a challenging environment. This challenge can be physical, but it can be cognitive and affective-emotional as well. We use the latter term as a general term without elaborating in depth on the distinction between *feelings* and *emotions*, as coined by Damasio [59,60]. His main claim is that the former are mental experiences of a bodily state, which arise when the brain interprets emotions, whereas the latter are physical states that arise from the body’s responses to external stimuli. Emotions, in his view, are a collection of complex unconscious neural responses that are representative of the body’s internal state and that cause observable external changes in the organism, which give rise to feelings. His theory is somewhat reminiscent of the controversial James–Lange theory of emotions, which has been criticized for favoring biological reductionism. Yet, it opens up the possibility of placing both emotions and feelings in an evolutionary perspective that considers their biological role in homeostasis, as well as their role in the mechanisms of survival.

Listening, in that broader conception, can be seen as coping behavior that can be defined beyond a conception of a mere tool for survival into a role for musical sense-making and reward [43]. As such, it involves overt physical reactions as well as mental and cognitive operations which are grounded in our biological functioning. This is an approach that argues for a continuity between environmental or natural sounds and musical stimuli, rather than conceiving of them in terms of a qualitative distinction, as already advocated by pragmatic philosophers such as Dewey and James [61,62] (see also [63,64]). Dewey, in particular, has argued for a definition of having an experience as a kind of heightened vitality with adaptive value, as exemplified in the life of the savage man who is in danger in a threatening environment. Observation, in that case, is not merely a way of gathering information for delayed or remote usage, but functions as a sentinel for immediate thought, both as action in preparation and foresight for the future [61] (p. 48).

Music listening, in this naturalistic view, entails the management and regulation of attention and arousal, somewhat analogous to the mechanism of coping with stress. Attention, in a coping context, has an explorative function, as a kind of open monitoring, but one that is fueled by arousal, which, in its most general definition, is a state of the brain or the body that reflects heightened responsiveness to sensory stimulation. It may range from low levels, as in calmness or boredom, to high responsiveness, as in anger or excitement. As a rule, it is correlated with increases in behavioral, hormonal, and/or neurological activity [65]. Applied to music, this should mean that music can be experienced either as a stressor or a reward, with the related concepts of adaptive and maladaptive listening in the sense that stimuli are valued either as beneficial (adaptive) or harmful (maladaptive). The distinction, however, is not absolute, as heightened arousal can have survival value as well, even with beneficial effects in the short term (see below). There is, nonetheless, a danger of overstimulation with lasting effects on the homeostatic baseline physiological level-setting [66]. Music, therefore, can affect our biological systems, both in a temporary or a permanent way, and either in a beneficial or a harmful way. The question, still, is how to assess this in an objective and generalizable way.

Several research strands have been proposed in this regard: the study of musical reward, with emphasis on hedonic pleasure; the enjoyment of sad music; and the adverse effects of music as a possible stressor. They all have their specific neuroendocrine signature, though the search for their explanatory mechanisms has not yet been satisfactorily clarified. There is, as such, a need for mediation analysis to trace the possible connections between the music as an elicitor and the effects and responses of the listener, with the major question being whether music is the causal agent or just the mediator of these effects. There are, furthermore, two major approaches to studying these underlying mechanisms: the cognitive and the affective functions, which are grounded in our basic homeostatic regulation. Both approaches revolve around the mechanisms of pleasure and reward, the twin notions of valence and arousal, the affect-related consequences of music listening, and the role of affective regulation and visceral reactions to the sounds, which, together, have been studied in the context of homeostatic emotions [67,68].

The role of the mechanisms that give rise to music-evoked emotions and affects, first, cannot be overstated. They entail an entire system of bodily mapping in the brain and bodily changes and actions, as well as the interactions with the musical instrument or one own’s body, used as an instrument, and the patterns in the music [69] (p. 337). As such, they can be considered as a multiplicative function of the structural features of the music, the listener, the performer, and the context [70]. There is, as such, a lot of variability among individual listeners. Yet, it is possible to generalize a little and to distinguish some common descriptions that characterize the bulk of music-induced emotions and affects. They embrace feelings of sadness, being touched, and tenderness [71], as well as being moved [72,73,74,75,76,77]. Being moved, in particular, plays a major role in the enjoyment of tragic art. It is to be considered a mixed but predominantly positive emotion and has been linked to prosocial and social-bonding behaviors. This remains largely opaque to spectators, however, which means that there is a need of scientific analysis to bring this to the fore (see [51] for an overview). Much is to be expected here from the neurochemistry of musical emotions, given their special role as mediators between physiology, behavior, and surviving and flourishing in the world [78]. Of particular importance in this regard are the serum levels of key hormones, such as prolactin (PRL), oxytocin (OT), cortisol, and adrenocorticotrope hormone (ACTH), and their complex relation with the dopaminergic system. The latter, in fact, is known to function as an inhibitor of peptide hormones (PRL and OT), which means that many of the known effects of these pleasure-inducing hormones seem to decrease in the case of pleasurable experiences [9]. Their functioning, therefore, must be understood in the fine balance between the action of dopamine as an inhibitor and many other factors, such as the hypothalamic, systemic, and local factors that act as stimulators. There are, however, still many unresolved issues with regard to the precise tracing of the hypothalamic-dopaminergic pathways and their overlapping effects on the function of the pituitary gland [79].

The cognitive approach, on the other hand, has biological roots as well. It links the affective-emotional experience to the domain of musical sense-making by the modulation of subcortical responses by cognitive cortical control. This coupling has been demonstrated in the case of an aesthetic experience, where the mere realization that a stimulus is offered in an aesthetic content may change the nature of this experience. Cognitive assessment of the stimuli may discount them as inconsequential because they are recognized as being artificial or fictional, with a corresponding cortical inhibition of subcortical responses [10,80,81,82]. In addition to this inhibitory aspect, however, there is also the empowering aspect of cognitive mediation with anatomical evidence for stronger connections and integration between the mesolimbic reward circuit deep into the brain and those areas that are involved in high-level cognition. These neural mechanisms enable top-down processes to allow previous experience, knowledge, and meaning to mold the perception and interpretation of musical stimuli so as to make them pleasurable to hear [83,84]. Care should be taken, however, not to take a reductionist stance here as the cognitive approach is not merely about control. It involves appraisal as well as familiarity and attention as major sub-facets of the cognitive approach, and this includes a much broader range of neural correlation than mere subcortical responses.

### 2.3. The Role of Disposition and Active Engagement

As mentioned above, it is possible to conceive of a naturalistic approach to the musical experience in terms of the mathematical analogy of a function or equation, with, on the one hand, a variable we can operate upon (the independent variable) and which can be considered as a sort of cause, and, on the other hand, a resulting change to another variable as its effect (the dependent variable). In addition to these variables, a mathematical equation may also contain constants, which do not change their values when the variables change and which may have a decisive role in the final outcome. These constants can be considered in an additive or multiplicative way, in the sense that the dispositional machinery of each musical listener—which we define here as the constants—provides a kind of basic level-setting that has an effect on the function from the very start of listening. It seems plausible to conceive of enduring personality traits, which are determined to a substantial degree by genetic factors and which stay relatively stable across the life span as individual differences between people, and to consider these dispositional factors as constants rather than as personality variables. In addition to these stable traits, however, there are dynamic factors, such as attention and/or motivation, which can increase or decrease the intensity of processing, either in a linear or a non-linear way and which can be considered as a proportionality factor that is responsible for the growth or attenuation of the processing in a real-time listening situation (see Figure 3 below for a full explanation). Yet, though intuitively appealing, the mathematical modeling of this analogy is extremely complex as both the innate disposition and the learning history of individual listeners are quite difficult to map. It can even be questioned whether we can conceive of a function in a strict mathematical sense here—i.e., a relation between two variables where every value of the independent variable is associated with exactly one value of the dependent variable—as there are many possible outcomes, given the wide variety of reactions and responses by the listeners. There are, however, some interesting approaches from the “individual differences” literature on personality and cognitive traits, with the Big 5 personality traits—Extraversion, Agreeableness, Conscientiousness, Emotional Stability/Neuroticism, and Openness to experience [85] and the role of trait empathy as major players in this regard.

Regarding the role of personality, underlying personality traits have been found to mediate physiological and psychological reactions to different styles of music [16,86,87,88,89]. Chills reactions, for example, seem to depend more on personality traits than on intelligence and background factors, even to the extent that it is possible to distinguish between chill responders and non-responders. The former can be associated more commonly with personality traits such as low sensation seeking and high reward dependence, as well as with musical preferences [90,91]. People who score high on openness to experience—defined as the breadth, depth, and permeability of consciousness with the aim to enlarge and examine the experience—and introversion also seem to experience aesthetic chills more frequently and claim to enjoy music-induced sadness more often [92,93,94]. Openness to experience, moreover, is associated with feeling comfortable with novelty and with motivation for cognitive exploration [95,96]. Other connections have been found as well, such as the likelihood of evoked sad feelings in listeners with a high score on agreeableness and neuroticism [97]. In neuroendocrine terms, this can be assessed by different levels of prolactin release and different degrees of susceptibility to the music’s consoling effects [10] though there is currently no conclusive evidence for the specific relation between increased or decreased levels of prolactin as related to the pleasurable or unpleasant music-induced sadness [9].

Empathy is another factor that may modulate the musical experience. Being defined as the ability to understand and feel what other people experience, it consists of two distinctive elements—an affective and a cognitive one—which can be delineated on the basis of behavioral, neuroanatomical, and neurochemical correlates. They involve involuntary emotional reactions, which are “evoked” by the observed emotions of others (affective empathy) or entail an intellectualized “recognition” or simple “understanding” of them without necessarily experiencing them themselves (cognitive empathy) [51,98,99,100,101]. Cognitive empathy, moreover, plays an essential role in perspective-taking, as in attributing mental states to others, and is linked to dopamine release. Affective empathy, in contrast, involves emotion recognition, emotional contagion, motor empathy, and shared pain, and is linked to the release of oxytocin [102]. Taken as a whole, trait empathy has been found to be a strong mediator of experienced pleasure, and this holds for the experience of sad music in particular [103,104].

It is a tedious and still unresolved question whether these dispositional traits are to be considered static and unchangeable traits—either innate or acquired—or as characteristics that are subject to adjustment and adaptation. Much is to be learned here from studies on neuroplasticity and brain connectivity as related to prolonged and intensive periods of musical engagement (see [105,106] for an overview). A distinction should also be made between engagement with a musical instrument as opposed to merely listening, in the sense that the former seems to develop neural and behavioral enhancements that are more pronounced than in the case of mere listening. The distinction between performing on an instrument and mere passive listening, however, is not radical. There are ways of engagement with music without instruments, such as dancing, clapping hands, singing with music, etc., and listening can be extremely active, while performing can be run even on auto-pilot as well, without real engagement.

Some effects of this engagement are traceable, even with adaptations seen on a very rapid time scale. It has led scholars to argue that music training can induce rapid cognitive and neural benefits, both in the audio-motor areas—in the case of active playing—and in the auditory cortical-evoked responses [107]. But still more impressive is the strengthening of anatomical connections between distinct areas of the brain, particularly with regard to the white matter connectivity, which seem to relate areas involved in hearing, emotional-affective processing, and moral-aesthetic valuing and judgments (see below). It clearly illustrates the viability and learnability of skillful listening as a learning path that involves both structural changes as well as the modulation of cognitive, emotional-affective, and even sensorimotor factors.

Summarizing a little, there is substantial variability among human listeners, both in the frequency and in the specificity of their aesthetic responses. Even if the neural circuitry for reward has been described in detail, what accounts for individual differences remains to some extent unclear. There are, however, new findings about white matter connectivity between auditory sensory processing areas (superior temporal gyrus) and emotional and social processing (medial prefrontal cortex, insula) which may explain the highly individualized differences in reward sensitivity to music. Listeners who frequently experience intense emotions and chills seem to have increased white matter connectivity among these three regions of the brain with observed differences in tract volume that arise from increased branching, differences in the width of the myelin sheath, and the higher structural integrity of those white matter pathways that overlap with major fiber bundles in the brain (arcuate fasciculus and uncinate fasciculus) [108]. The findings have clinical importance for differences in behavior between people with high emotional empathy and people with social–emotional impairments. Higher white matter connectivity has been observed for the former, whereas lower white matter connectivity was observed in people with social–emotional impairments, mood disorders, and schizophrenia [109,110,111,112]. It seems, moreover, that people who have difficulties in experiencing strong emotional responses to musical stimuli should also be susceptible to insensitivities or impairments in emotional and social functioning [113].

## 3. Rewards of Music Listening

Recent research has focused largely on the effects of pleasure associated with music and has shown that music activates neural circuits that are involved in emotion and reward [13,84,107,114,115,116,117,118]. Music listening, in this view, can be considered as the experience of feelings of hedonic pleasure and displeasure in combination with some degree of arousal, mediated through the visceral and peripheral sensory systems, and some mental representation of these bodily changes. Or, put differently: the musical experience revolves around valence and arousal, which can be considered as the basic dimensions of core affect [119,120].

### 3.1. Music and Pleasure: The Role of Endogenous Opiates

There exist different views on reward, which revolve around the hedonic psychology of pleasure and pain (see [83] for an overview), with links to behavioral theories of reward and punishment as well as theories of cognitive expectations about possible outcomes [3,121]. The latter fit with an expectancy–value approach which states that well-being is a function of the expectation to attain valued outcomes [122,123]. So, there seems to be a kind of conceptual divide between the mere sensory level of hedonic pleasure and its cognitive valuation. The first, also coined as the sensory hypothesis of musical pleasure, focuses on the sensory mechanisms that determine the succession of neural events from the periphery of the body to the central nervous system. It is possible, however, to go beyond this sensory processing by soliciting cognitive mechanisms that allow a transition to a conscious hedonic feeling of liking through the mediation of higher-order structures in the brain. This is the conceptual hypothesis of musical pleasure [120], which implies psychological processes of cognitive mastering as a crucial stage of information processing that leads to aesthetic outcomes of judgments and emotions [124,125,126].

Both approaches have received empirical evidence from neuroaesthetic research with, as an interesting contribution, the neural chronometry of the aesthetic experience, which breaks down the processing into several distinct stages [120,127]. There is, first, an initial, mainly unconscious stage of neural processing of emotional stimuli, which can be viewed as early affective reactions to the music, somewhat similar to core affect. These first reactions are followed by value attributions, which originate from prefrontal and associative cortices, and which modulate these early responses to become conscious emotions such as sadness, happiness, and conscious enjoyment. This can be done by the mediation of personal associations, previous knowledge, social constructs, or other top-down processes that require language, executive attention, episodic memory, and categorization processes.

The picture that comes up here points in the direction of an intense interplay between cortical and subcortical structures in the sense that the mechanisms that are involved in fundamental pleasures, such as food and sex, are also found to overlap with higher-order pleasures, such as monetary, artistic, musical, prosocial, and transcendent pleasures [118,128,129,130,131,132]. They all seem to involve the same hedonic brain systems that evolved for sensory pleasures and that are distinct from the mediation of other features, such as the sensory or cognitive ones [133].

This brings us to the neuroendocrinology of musical reward, with a special focus on the working of the dopaminergic reward system [83]. Dopamine is a crucial neurotransmitter in the reward system, and intensely pleasurable responses to music have been found to co-occur with dopaminergic activity in the striatal system—a small group of subcortical structures which are part of the basal ganglia and which include the caudate nucleus, the putamen, and the nucleus accumbens [134] (p. 953). Furthermore, the study of dopamine release in different brain regions while listening to emotionally evoking music has revealed an unforeseen functional dissociation between two involved anatomical pathways that play different but complementary roles: the caudate nucleus seems to show increased activity during the anticipation of peak emotional experiences; the nucleus accumbens, on the other hand, is associated with dopaminergic activity during the actual experience [84,135].

The distinction is exemplary of the complex nature of the underlying neuroendocrine mechanisms of musical pleasure. There is, for the time being, no conclusive evidence with respect to the neurochemical indicators of reward, particularly with respect to the concentrations of prolactin and oxytocin and their relation to the release of dopamine. There is, however, considerable agreement on the major role of the nucleus accumbens in controlling the release of dopamine. Its functioning is associated with processes of reward, pleasure, and motivation and points in the direction of primary activities for survival [70]. The underlying mechanisms, however, are not yet totally clear, with distinct but opposed theoretical constructions, such as the “reward theory” against the “homeostatic theory” [9].

The homeostatic theory assumes that hormonal changes reflect a homeostatic function of neutralizing the negative effects that social distress and loss can trigger [10]. It predicts an increase in the prolactin level in response to possible stressors and an increase in the oxytocin level in relation to its anxiolytic function [11,12,136,137]. This increase in prolactin at a proximal stage is hypothesized to lead to reward at a distal stage. Empirical testing of the theory in the case of exposure to sad music, however, did not yet corroborate these findings. The reward theory, in contrast, states that psychological reward and its neurochemical correlates originate from the dopaminergic system that is involved in prediction and anticipation [14,15]. Combined psychophysical, neurochemical, and hemodynamic measurement procedures have revealed that dopamine release in the nucleus accumbens is associated with peaks of autonomous nervous activity that reflect the experience of an intense emotional moment. This mechanism has been found to engage music-induced pleasure at a proximal stage, which points in the direction of the role of dopamine as a causal factor in the hedonic experience that is induced by music [13]. There is, in sum, neurochemical evidence that music has the potential to manipulate hedonic states by involving the ancient reward circuit, with large interconnections between limbic regions, such as the amygdala, hippocampus, cingulate cortex, and prefrontal cortex, which all mediate emotional responses [135].

The role of prolactin, however, is less clear, as its release is controlled by the dopaminergic system, with dopamine itself being known as an inhibitor of endogenous prolactin release [79,138]. This means that pleasurable activities which produce dopamine should decrease the release of prolactin. These findings, however, are at odds with previous theoretical positions regarding the hedonic theory of music-induced sadness, which can be experienced either in a positive or a negative sense. It is hypothesized, in this view, that levels of prolactin increase when people are feeling sad so as to produce a consoling psychological effect, which is suggestive of a homeostatic function. Variations in prolactin levels, moreover, should account for the variability in the hedonic responses of individual listeners with the conjecture of high concentrations of prolactin in the case of pleasurable music-induced sadness and low levels in the case of unpleasant sadness [10]. The findings, however, have not yet been corroborated by empirical research—there is in fact growing evidence against them—and remain the object of heated discussions [51,139,140]. It may be questioned, in this regard, whether the individual profiles of listeners and the role of their evaluative weightings could provide a possible solution to this not-resolved paradox.

The effects on oxytocin, moreover, are also not totally clear. This neuropeptide, which is involved in social behavior and rewarding behaviors, is assumed to interact with the dopaminergic system to enhance attention towards social stimuli and thus increase their rewarding potential [141,142]. The effect on oxytocin levels after positive emotion induction, however, has yielded mixed patterns of results, with both increased and decreased levels of oxytocin. It is, in fact, such a complex topic, with implications in a wider variety of functions, as to resist simplified explanations and reductions to a linear causality (see [143,144] for interesting overviews). It also means that the neuroendocrine underpinnings of musical pleasure, though extremely challenging, are still inconclusive at this moment.

### 3.2. Aesthetic Experience and Reward: Peak Experiences, Chills and Thrills

People value music primarily for aesthetic reasons, for the emotions it generates, for the memories it can trigger, and for its perceived beauty [145]. Listening or performing, moreover, can generate aesthetic experiences, which include specific emotions and evaluative judgments of beauty, aesthetic quality, and liking [70]. It can be questioned to what extent such aesthetic experiences can be generalized to the domain of heightened affective experiences or peak experiences, which have been defined as intense psychological states with feelings of the highest happiness and fulfillment and which are characterized by enhanced attentiveness and considerable degrees of absorption and immersion in the eliciting activities [146,147,148].

An interesting methodological approach, in this regard, is the assessment of measurable bodily reactions as the outcome of peak emotional responses to music, such as goosebumps or shivers down the spine, commonly referred to as chills. They rely on dopaminergic neurotransmission, triggered by the hypothalamus, which is able to modulate the sympathetic and parasympathetic activity of the autonomic nervous system via brainstem responses that regulate physiological responses, such as heart rate, blood pressure, body temperature, skin conductance, and muscle tension [89] (p. 186). All these responses can be measured continuously and objectively. They involve a clear and distinct activation pattern of intense autonomic nervous system arousal, which is hypothesized to underlie peak pleasure during music listening [114,149,150]. As such, they provide a much-valued objective index of subjective pleasure by providing empirical support for intensive emotional reactions to the music. In addition, they also make it possible to pinpoint the precise time moments of perceived maximal pleasure [135].

Chills, however, do not show the simple linear stimulus–response pattern that was hoped for, as listeners seem to react to musical patterns and meanings rather than to mere acoustical triggers. The way they make sense of acoustic stimuli, in fact, is modulated by their musical preferences and individual learning histories [91]. There is, as such, not yet a conclusive explanation of how these aesthetic chills are elicited: there are lots of varieties of eliciting musical features, as well as distinct and even conflicting theoretical constructs. Major questions concern the psychological constructs of peak pleasure, chills, and thrills, and whether these should be regarded as unified constructs or as sets of distinct categories of responses that may vary in terms of actual experience, their elicitors, and the individual differences among chill-responders [151].

As a first attempt to analyze these terms, it is possible to provide a distinction between thrills and chills: thrills are linked mostly to novelty or a new-found insight, with accompanying feelings of tension, awe, or sublimity; chills, on the other hand, are related mainly to absorption and being moved, resulting in peak emotional experiences [151,152]. The distinction, however, though useful, is not always respected in much empirical research, with both terms being used interchangeably.

The psychological significance of thrills, in general, has already received a lot of exploratory work. They are considered to be the most common and the least differentiated aesthetic responses of the so-called aesthetic trinity, which encompasses thrills, the state of being moved, and the experience of awe [153]. They include a family of unusual states, such as aesthetic chills, feeling touched and moved, losing track of time, feelings that resemble crying, the experience of awe, and absorption and detachment from the surroundings [93]. The aesthetic chills, in particular, have been widely studied, with the identification of distinct chills constructs on the basis of affective valence, the qualities of the eliciting stimuli, and individual differences among the chills-responders. Experimental analysis of bodily reactions, moreover, has revealed three dimensions of chills experience, characterized, respectively, by (i) frowning, smiling, and feelings of warmth or cold; (ii) tingling, shivers, and goosebumps; and (iii) tears and feeling a lump in the throat [151]. Combining these bodily reactions with emotional experience makes it possible to construct three distinct categories of chills: warm chills, which reflect positive feelings as joy, stimulation, relaxation, and feelings of warmth; cold chills, reflecting negative feelings; and moving chills, characterized by bodily symptoms such as tears and a lump in the throat, feelings of tenderness, affection, intensity, and being moved [151].

The study of aesthetic chills, however, still faces methodological problems. There is, first, the distinction between chills, experienced as a subjective phenomenon, and their objective assessment, with the attempts to bring them together by conceiving reported bodily activity as the main dependent variable, and the rating of subjective feelings as supplementary variables [135,151]. There are, second, the different research traditions that approach aesthetic chills from the experimental side—how features of the music affect listeners (see [154,155,156] for a study on the contribution of causal manipulation of features of the music on the experience of chills) and how physiological parameters correspond to chills experiences [91,114] or from the individual difference side (see [93] for an overview). The latter has received less attention than the experimental approach, though there are provisional findings that openness to experience could be considered a cross-cultural marker of aesthetic chills [92]. It has also been found that the experience of chills is related to the feeling of control, in the sense that people who experience chills are inclined to listen to music that has a special meaning for them and which is able to evoke strong emotions, both in a positive and a negative sense [93]. More generally, it can be stated that chills experiences are sought after by a specific population, to function as a hedonic experience regardless of the affective valence [151]. There is, third, the scope and breadth of chills research, with a traditional orientation towards positive chills. Yet, there are contexts in which listeners can also experience negative chills [151,157] and which highlight the possible role of musical stimuli as possible stressors, which can be valued in a positive or negative sense, either at a physical level of distress or at the level of communally shared emotions with regard to negative stimuli. The above-mentioned cold chills point partly in this direction, as well as the research into being moved, with a major distinction between being joyfully or sadly moved [151,158,159].

There are, finally, also new strands of research that try to expand the current explanations of chills, linking the experience of chills to musical features, psychophysiological activity, and individual differences by distinguishing between vigilance chills and social chills. The former are linked to awe, expectancy, and auditory looming; the latter are linked to being moved, empathy, and social bonding [160].

## 4. Listening beyond the Sounds: Eudaimonic Pleasure

Some elementary perceptual features of sounds may produce hedonic sensations by themselves, with the aesthetic enjoyment of music being derived in a bottom-up manner from the pleasure that originates from the acoustic features of the sounds. This is the “sensory hypothesis” of musical pleasure, which relies on neural mechanisms that are grounded in features of evolutionary and behaviorally relevant animal sounds [120,161]. Empirical research within the field of hedonic psychology has revolved largely around this bottom-up approach, with a focus on pleasure and pain [3] (but it also has pointed to the distinction between those experiences that are merely pleasurable and those that are enjoyable. The former involve the satisfaction of homeostatic needs; the latter involve those feelings that go beyond the constraints of homeostatic functioning [1]. Enjoyment, therefore, is what leads to personal growth and long-term happiness. It transcends mild dysphoria to produce feelings of subjective well-being, including components of life satisfaction, the presence of positive mood, and the absence of negative mood, all of which can be summarized under the umbrella term of happiness [162], and which point in the direction of a full-fledged musical experience.

### 4.1. From Sensory Pleasure to Eudaimonic Enjoyment

There are two major conceptions of happiness, termed hedonic enjoyment and eudaimonia, with pendants in either the hedonic or the eudaimonic schools in hedonic psychology [3,163,164].

The hedonic perspective revolves around the subjective evaluation of the quality of life. It reflects the view that well-being consists of the experience of pleasure or happiness, with a focus on the experience of pleasant feelings, a balance between positive and negative affect, and the cognitive–affective measure of life satisfaction. It has been defined more concisely as “more positive affect, less negative affect, and greater life satisfaction” [3,165]. This centrality of hedonic well-being or enjoyment is oriented primarily toward being relaxed, avoiding problems, and being happy [162]. It goes back to Bentham’s view of pleasure as the only thing that is good for us and that considers pain as a bad thing to be avoided. It is important, however, not to confuse hedonic happiness with hedonism, as the latter aims at the pursuit of pleasure merely for its own sake, as is the case with addiction (see below) [166,167]. Such a narrow conception of happiness as hedonism, therefore, has gone out of vogue in an attempt to include a broader range of positive and negative feelings and emotions beyond pleasure and pain, which were at the center of psychological theories toward the end of the 19th century [2]. Yet, the potential contributions of hedonics to happiness have seen a revival in the study of the brain mechanisms of pleasure, which are present and similar in most mammalian brains. As such, they seem to ground what is now considered a “science of pleasure” [167,168].

The eudaimonic perspective, on the other hand, focuses on psychological well-being and states that well-being consists of more than mere happiness by stressing the role of full functioning [169]. It is a conception, inspired by Aristotle, that triggers people to live in accordance with their daimon or true self—hence the term eu-“daimonic”—to feel intensely alive, authentic, and holistically or fully engaged so as to actualize their human potential. It means that their activities should be congruent with deeply held values so as to have a feeling of existing as a personal expressiveness of who we really are [170]. Eudaimonia, therefore, measures the extent to which people are doing well, rather than merely feeling good. It involves a broader and more holistic view than the hedonic position and is more related to being challenged and exerting effort, stressing the actualization of human potentials by engaging in diverse experiences and mechanisms to make meaning and to seek a purpose in life [2,164]. As such, it can be considered happiness with meaningfulness [171].

The mutual relation between hedonic happiness and eudaimonic enjoyment is very complex. It is even unclear how pleasure and happiness are exactly linked, though considerable progress has been made in the understanding of the neurobiology of pleasure. The hedonic approach, at least, provides a starting point for the identification of the eudaimonic brain signatures of happiness [166,167]. On the negative side, it has been found, for example, that pathological conditions of lack of pleasure, such as anhedonia or dysphoria, seem to function as major obstacles to happiness. Hedonic happiness, furthermore, can be a sufficient but not necessary condition for eudaimonic enjoyment, given the multiple routes to hedonic happiness beyond engaging in personal expressiveness. As such, four categories of feelings that arise in connection with particular activities can be identified: those that give rise to both eudaimonia and hedonic enjoyment; those that are hedonically enjoyed without giving rise to eudaimonia; those that are neither hedonically enjoyed nor give rise to eudaimonia; and those that give rise to eudaimonia but without being enjoyable in the hedonic sense of the term [172,173]. Generalizing a little, it can also be stated that the mechanisms which are involved in fundamental pleasures overlap with those for higher-order pleasures: pleasures related to happiness and positive hedonic mood all draw upon the neurobiological roots that evolved for sensory pleasure. They all involve the same hedonic brain systems, which are distinct from other systems that are involved in sensation and thought [167]. It can be stated, therefore, that well-being, as a broader category, can be best conceived as a multidimensional phenomenon that embraces both hedonic and eudaimonic aspects [3]. The question remains, however, whether both perspectives should be considered as orthogonal categories or as categories that are intertwined to some extent, with the related question of whether they can be studied separately. The answer is probably to be found in a reliance of distinct levels of processing with a dynamic tension between the bottom-up and top-down approaches.

### 4.2. Listening to Music for Self-Reflection and Self-Realization

Music is not merely about sounds. It can trigger a lot of chemical and neural reactions at a proximal stage which may give rise to emotional responses and valuing at a more distal stage that are more distant from low-level processes. As such, it may operate as an emotionally competent stimulus—to use Damasio’s term [60] (p. 53)—that allows listeners to react with a specific action repertoire, which may result in a change of the state of the body so as to create circumstances that are optimal to survival and well-being. This holds in particular for positive emotions in the appraisal of music, which have been found to broaden the listener’s behavioral and cognitive repertoire. It is a conjecture of the Broaden-and-Build Theory [174], which states that positive emotions did evolve to consolidate and expand resources by attempting more creative courses of action. The result is a broadening of the scope of attention, an expansion of thought–action repertoires, an increased openness to new experiences, and a readiness to engage in holistic processing, with the ultimate aim of building long-term physical, psychological, intellectual, and social resources such as resilience and curiosity (see [2] for an overview).

The experience of positive emotions can also be related to the field of optimal functioning within personality psychology. It has been explored in the context of well-being and human flourishing and has been theoretically and operationally defined as psychological well-being, which embraces six aspects of human actualization that specify emotional and physical health: autonomy, personal growth, self-acceptance, life purpose, mastery, and positive relatedness [169]. Revolving around the theoretical constructs of personal identity, self-actualization, internal locus of control, and principles of moral reasoning, it shares most of the foundational claims of eudaimonia [170].

It is tempting to apply this to the realm of music, which can be seen as having the power to affect the quality of life through emotion regulation and observable effects on both behavior and brain functioning [175]. Music, in this view, can be used for self-reflection—an ability that requires internally directed cognition—as seen most typically in the case of listening to sad music, which is considered by some as a major source of enjoyment [9,10,51,103,104,140,176,177,178,179,180]. Listening to sad music, moreover, has also been put in relation to the phenomenon of depressive realism, which states that people are more realistic when they are sad. Sadness, as compared to happiness, is likely to provide a kind of mental grounding and a reality check by encouraging more detail-oriented thinking, fewer judgment biases, and a more realistic assessment of the likelihood of certain outcomes [181,182,183]. Listening to sad music, accordingly, may induce such depressive realism, with more accurate self-appraisal as a positive effect [10,184].

## 5. Adaptive and Maladaptive Listening

Curiosity and openness to new experiences can trigger the search for pleasurable and enjoyable experiences. A distinction should be made, however, between enjoyment and pleasure, in the sense that pleasure is oriented primarily towards the mere satisfaction of homeostatic needs, whereas enjoyment can break through these constraints [1]. The search for pleasure, however, espouses the centrality of hedonic well-being with an emphasis on the balanced equilibrium between positive and negative affect. As such, it has become a major part of affective neuroscience, which studies the neural mechanisms that underlie the generation of affective reactions in humans and animals in an attempt to identify the objective features of pleasure reactions—with measurable aspects in the behavioral, physiological, and neural reactions—in addition to the more subjective experiences of conscious affective feelings [167,168]. A major finding has been the adaptive nature of positive and negative affect, particularly with regard to planning and building cognitive and emotional resources [185,186,187].

Music, in that view, can be used as a tool for aesthetic empowerment. Some tentative neurobiological mechanisms have been proposed in this regard, with possible beneficial effects, such as improved mood, focused attention, facilitation of learning, and the enhancement of memory [134,188]. The need to enhance mood and motivation has, in particular, generated a lot of empirical research with a focus on the role of dopaminergic activity within the reward circuit. Some findings are rather obvious: enhanced emotional processing through autonomic activation seems to trigger an elementary and spontaneous core liking reaction that is generated in the hedonic hotspots of the pleasure system, even when a conscious feeling of liking does not occur. This activation is accompanied also by “wanting” or “incentive salience” during the appetitive phase of the experience [189]. Besides these core dopaminergic reactions, there is also the related physiological mechanism, which is known as sympathetic arousal. Due to dopaminergic neurotransmission and anatomical connections with the hypothalamus, the emotional responses to music can modulate the sympathetic and parasympathetic activity of the autonomic nervous system, thus triggering physiological responses which can regulate the listener’s mood [89].

It can be questioned to what extent these neurobiological mechanisms are adaptive in a beneficial sense. There is, in fact, the possibility of adaptive and maladaptive listening with listening behaviors that may possibly be related to maladaptive response styles [43,190]. The latter might be the case for listening to music for mood regulation when there is no real enjoyment of the music or when no psychological benefits are obtained from listening [191,192]. There exist, after all, maladaptive motivations for listening, especially with regard to sad music [94,193]. It is possible, however, that people enjoy the pure hedonic pleasure of negative emotions, or that they are attracted to it for some psychological benefits.

Crucial in these motivations is the coupling of cortical with limbic regions—the mesocorticolimbic pathway—which points in the direction of a larger, general-purpose system that enables a seeking disposition towards the environment to establish adaptive expectations about its configurations and availability for reward [194]. Findings regarding dopamine release have shown that the seeking disposition itself may have hedonic properties, irrespective of the actual attainment of reward, with a distinction between tonic dopamine release to maintain a baseline level in the brain and phasic bursts that fire in response to specific cues. Examples of the latter are unpredicted rewards, prediction errors, novel stimuli, physical, motivational, or affective salience, and attention shifts that are related to approach behavior. Tonic release, on the other hand, promotes arousal in most mammals [195].

It is interesting, in this regard, to explore the possible analogy between music consumption and addictive behavior. Addiction tends to strive for too much wanting. It is related to the mechanism of medium maximization, where one loses sight of the ends of utility to focus instead on the means (see [2,196] for an overview), with the potential danger of reducing hedonic happiness to mere hedonism, which can be seen as the pursuit of pleasure for its own sake.

This pursuit of medium maximization has its counterpart in the management of arousal, with its corresponding levels of stress, which can be perceived either as being harmful/annoying or beneficial for better coping behavior. It is typical of personality traits such as sensation seeking and a desire for rebelliousness, which, in the case of music, may influence the appreciation of loud sounds, which are valued for providing intense stimulation and arousal [197,198].

Such overstimulation can lead to patterns of addiction and maladaptive listening [191,192] with the pathological usurpation of those neural processes that, in normal conditions, serve reward-related learning. They involve such structures as the nucleus accumbens, the ventral tegmental area, the dorsal striatum, and the prefrontal cortex [199]. The actual impact of loud sound, however, is difficult to control, as its enjoyment depends on complex and powerful interactions of cultural, interpersonal, and interpersonal factors, which all point towards an ecology of acceptance of high-level sound (see [200] for an overview).

It makes sense, therefore, to argue for ways of listening that attune listeners to sonic environments—including music—that provide stimulation in the optimal arousal of arousal, to cultivate positive adaptive reactions to beneficial stressors, as well as to avoid the possible distress that may potentially be triggered by harmful stimuli [193,201].

Adaptive listening, in sum, should not be defined solely in terms of the avoidance of harmful stimuli. There is some analogy, here, with the definition of health, which was originally conceived solely in terms of the medical model with its focus on reducing disease and disability. Recent definitions of health, in contrast, have broadened its scope by also giving attention to the nature of health and well-being [163]. In the same vein, it can be argued that adaptive music listening should not limit itself to going beyond the pathological lack of pleasure, as in anhedonia or dysphoria, or to the avoidance of maladaptive patterns, as in addiction. It should aim instead at reaching the level of aesthetic enjoyment which is characterized by a potential interaction with eudaimonic networks. This is a claim that links pleasure or positive affect to happiness, with a lot of empirical grounding from the neurobiology of pleasure.

## 6. Conclusions and Perspectives

In this paper, we have tried to elaborate on the possible relation between music as a stimulus and its possible effects, with a focus on enjoyment and reward. Though it is tempting to seek for some linear causal relationship, this has proven to be elusive to some extent, given the many intermediary variables that intervene between the actual impingement on the senses and the reactions/responses by the listener. A promising approach, in this regard, is to start from Tinbergen’s distinction between proximate and ultimate stages of explanation. The former can be described in terms of biochemistry and physiological and neural correlates and can help explain why some mechanisms work at all (mechanistic explanations) and how they developed over an organism’s lifetime (ontogenetic or developmental explanations). The latter are oriented towards longer time scales and raise questions about the evolutionary history of acquisition and modification of a trait (phylogenetic development) and its ultimate function or survival value. Proximate stages are the domain of physiology and developmental biology; ultimate stages are components of modern evolutionary biology (see [40] for an overview). Both levels of explanation are complementary, but the proximate stage has a special role in providing the much-needed arguments for naturalizing the musical experience in an attempt to describe and explain it in terms of empirical evidence. It makes it possible to align the study for musical enjoyment and reward with the “science of pleasure” and to gratefully use the findings from current neurobiological and psychobiological research.

Care should be taken, however, to avoid the danger of reductionism. As stated throughout the paper, music listening is much more than the mere sensory processing of the sounds. There is, moreover, the distinction between hedonic pleasure and eudaimonic listening, with lots of theoretical frameworks and explanatory mechanisms which are not yet conclusive at this stage. What is needed, therefore, is a more encompassing framework that starts from the mere biological levels of coping with the sounds, to affect and impact ever higher levels, such as the affective-emotional and the cognitive and mental level of processing. The latter, though being of utmost importance, has not yet received sufficient attention from the scientific community.

Music listening, in our broadened view, is to be considered as a multivariate phenomenon of biological, psychological, and cultural factors that, together, shape the ultimate experience. It is stated, furthermore, that listening may entail both adaptive and maladaptive ways of coping with the sounds. The search for musical reward, therefore, should not be the ultimate aim of listening, as this may lead to medium maximization and the search for overstimulation, with the possible danger of addictive behavior. What we argue for, is an adaptive way of listening that allows a gentle transition from mere hedonic pleasure to eudaimonic listening. The latter, however, is still waiting for additional and more substantial empirical support, though the recent findings from neuroscience are quite promising in this regard. In particular, the neuroplastic findings from increased white matter connectivity in the case of aesthetic listening and the found relationship between the aesthetic experience and moral decision making seem to open new perspectives for future research. They seem to corroborate the beauty-is-good stereotype and the old adage that music softens the morals.

It should be mentioned, however, that most of this research has been done with Western participants. Opening the scope of research to non-Western music cultures and practices might be useful in highlighting which aspects are culturally determined [202].

## Data Availability

Not applicable.
